# Supplementary material for: Model-Free Approach for the Configurational Analysis of Marine Natural Products
Source: Mar Drugs. 2021 May 21;19(6):283. doi: 10.3390/md19060283 (PMC8223791; doi:10.3390/md19060283)
Supplement: Supplementary file 1 [file marinedrugs-19-00283-s001.zip › marinedrugs-1196481-supplementary.pdf]

Supporting Information for

Model-free Approach for the Configurational Analysis of Marine Natural Products

Matthias Köck[a], Michael Reggelin[b], and Stefan Immel[b]

[a] Alfred-Wegener-Institut für Polar- und Meeresforschung in der Helmholtz-Gemeinschaft, Am Handelshafen 12, 27570 Bremerhaven, Germany

[b] Clemens-Schöpf-Institut für Organische Chemie und Biochemie, Technische Universität Darmstadt, Alarich-Weiss-Straße 4, 64287 Darmstadt, Germany

Table of Contents

Formulas and Atom Numbering of Compounds 1a-c, 2 and 3 1

NOE and Structure Data for Palau'amine Derivatives (1a-c) 2

NOE and Structure Data for Plakilactone H (2) 3

NOE and Structure Data for Manzamine A (3) 4

Atomic Coordinates for Compounds 1a-c, 2 and 3 5

Formulas and Atom Numbering of Compounds 1a-c, 2 and 3

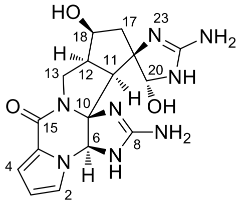

1a

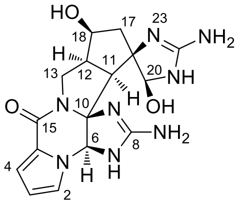

1b

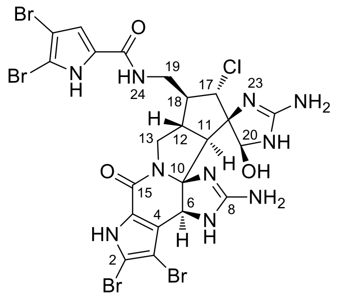

1c

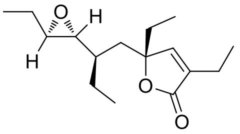

2

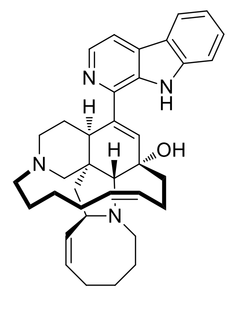

3

NOE and Structure Data for Palau'amine Derivatives (1a-c)

The following Tables S1a-c and Figure S1 show the experimental and back-calculated NOE data used for the configurational analysis of the palau'amine derivatives **1a**-**c**. The Tables were generated directly from the output files for the *ConArch*$^{+}$*/DG* best-fit (minimum pseudo energy) structure obtained from configurational and conformational analysis.

Table S1a. NOE data used for **1a**. The experimental data is listed as d_{mean}, and the allowed lower and upper bonds – here $d_{mean} \pm 10\%$ – are labeled as $d_{lower} \cdots d_{upper}$; the averaged distances back-calculated from *ConArch*$^{+}$*/DG* best-fit (minimum pseudo energy) structure data are labelled $d_{averaged}$, and the corresponding residuals are listed only if this value falls out of range, i.e. $d_{averaged} < d_{lower}$ or $d_{averaged} > d_{upper}$; all distance are given in [Å].

	D(averaged)	Deviation	(Percent)	D(lower)	D(mean)	D(upper)	(+/-Range)	Weights	NOE Contact
NOE[01] =	2.723941	-	(-)	2.36700	2.63000	2.89300	(10.0%)	1.000000	H5 || H6
NOE[02] =	2.616689	-	(-)	2.16900	2.41000	2.65100	(10.0%)	1.000000	H6 || H11
NOE[03] =	2.246777	-	(-)	1.98900	2.21000	2.43100	(10.0%)	1.000000	H11 || H12
NOE[04] =	3.203849	-	(-)	3.01500	3.35000	3.68500	(10.0%)	1.000000	H11 || H13B
NOE[05] =	3.357410	-	(-)	2.76300	3.07000	3.37700	(10.0%)	1.000000	H11 || H17B
NOE[06] =	3.011915	0.041915	(11.6%)	2.43000	2.70000	2.97000	(10.0%)	1.000000	H11 || H20
NOE[07] =	2.908557	-	(-)	2.57400	2.86000	3.14600	(10.0%)	1.000000	H12 || H13A
NOE[08] =	2.297098	-	(-)	2.03400	2.26000	2.48600	(10.0%)	1.000000	H12 || H13B
NOE[09] =	2.506087	-	(-)	2.50200	2.78000	3.05800	(10.0%)	1.000000	H12 || H17B
NOE[10] =	2.310568	-	(-)	2.24100	2.49000	2.73900	(10.0%)	1.000000	H12 || H18
NOE[11] =	1.766449	-	(-)	1.60200	1.78000	1.95800	(10.0%)	1.000000	H13A || H13B
NOE[12] =	3.177942	-	(-)	2.81700	3.13000	3.44300	(10.0%)	1.000000	H13A || H18
NOE[13] =	1.794118	-	(-)	1.60200	1.78000	1.95800	(10.0%)	1.000000	H17A || H17B
NOE[14] =	2.694828	-	(-)	2.34900	2.61000	2.87100	(10.0%)	1.000000	H17A || H18
NOE[15] =	2.477978	-	(-)	2.15100	2.39000	2.62900	(10.0%)	1.000000	H17B || H18
NOE[16] =	2.206491	-0.007509	(-10.3%)	2.21400	2.46000	2.70600	(10.0%)	1.000000	H17B || H20

Table S1b. NOE data used for **1b**. The experimental data is listed as d_{mean}, and the allowed lower and upper bonds – here $d_{mean} \pm 10\%$ – are labeled as $d_{lower} \cdots d_{upper}$; the averaged distances back-calculated from *ConArch*$^{+}$*/DG* best-fit (minimum pseudo energy) structure data are labelled $d_{averaged}$, and the corresponding residuals are listed only if this value falls out of range, i.e. $d_{averaged} < d_{lower}$ or $d_{averaged} > d_{upper}$; all distance are given in [Å].

	D(averaged)	Deviation	(Percent)	D(lower)	D(mean)	D(upper)	(+/-Range)	Weights	NOE Contact
NOE[01] =	2.685759	-	(-)	2.35800	2.62000	2.88200	(10.0%)	1.000000	H5 || H6
NOE[02] =	2.428717	0.008717	(10.4%)	1.98000	2.20000	2.42000	(10.0%)	1.000000	H6 || H11
NOE[03] =	2.396746	-	(-)	2.05200	2.28000	2.50800	(10.0%)	1.000000	H11 || H12
NOE[04] =	2.998957	-0.007043	(-10.2%)	3.00600	3.34000	3.67400	(10.0%)	1.000000	H11 || H13B
NOE[05] =	3.687766	-	(-)	3.09600	3.44000	3.78400	(10.0%)	1.000000	H11 || H17B
NOE[06] =	2.138654	-	(-)	1.87200	2.08000	2.28800	(10.0%)	1.000000	H11 || H20
NOE[07] =	2.820761	-	(-)	2.58300	2.87000	3.15700	(10.0%)	1.000000	H12 || H13A
NOE[08] =	2.358595	-	(-)	2.02500	2.25000	2.47500	(10.0%)	1.000000	H12 || H13B
NOE[09] =	2.614365	-	(-)	2.41200	2.68000	2.94800	(10.0%)	1.000000	H12 || H17B
NOE[10] =	2.326254	-	(-)	2.04300	2.27000	2.49700	(10.0%)	1.000000	H12 || H18
NOE[11] =	3.096064	-	(-)	2.54700	2.83000	3.11300	(10.0%)	1.000000	H12 || H20
NOE[12] =	1.757781	-	(-)	1.60200	1.78000	1.95800	(10.0%)	1.000000	H13A || H13B
NOE[13] =	3.075460	-	(-)	2.62800	2.92000	3.21200	(10.0%)	1.000000	H13A || H18
NOE[14] =	1.765112	-	(-)	1.57500	1.75000	1.92500	(10.0%)	1.000000	H17A || H17B
NOE[15] =	2.740695	-	(-)	2.40300	2.67000	2.93700	(10.0%)	1.000000	H17A || H18
NOE[16] =	2.410075	-	(-)	2.07900	2.31000	2.54100	(10.0%)	1.000000	H17B || H18
NOE[17] =	3.214872	0.024872	(10.9%)	2.61000	2.90000	3.19000	(10.0%)	1.000000	H17B || H20

Table S1c. NOE data used for **1c**. The experimental data is listed as d_{mean}, and the allowed lower and upper bonds – here $d_{mean} \pm 10\%$ – are labeled as $d_{lower} \cdots d_{upper}$; the averaged distances back-calculated from *ConArch*$^{+}$*/DG* best-fit (minimum pseudo energy) structure data are labelled $d_{averaged}$, and the corresponding residuals are listed only if this value falls out of range, i.e. $d_{averaged} < d_{lower}$ or $d_{averaged} > d_{upper}$; all distance are given in [Å].

	D(averaged)	Deviation	(Percent)	D(lower)	D(mean)	D(upper)	(+/-Range)	Weights	NOE Contact
NOE[01] =	2.431126	0.011126	(10.5%)	1.98000	2.20000	2.42000	(10.0%)	1.000000	H6 || H11
NOE[02] =	2.745016	-	(-)	2.25000	2.50000	2.75000	(10.0%)	1.000000	H11 || H13B
NOE[03] =	3.618106	0.021106	(10.6%)	2.94300	3.27000	3.59700	(10.0%)	1.000000	H11 || H17A
NOE[04] =	2.293836	-	(-)	2.09700	2.33000	2.56300	(10.0%)	1.000000	H11 || H18
NOE[05] =	2.992198	0.011198	(10.4%)	2.43900	2.71000	2.98100	(10.0%)	1.000000	H11 || H20
NOE[06] =	2.636674	0.007674	(10.3%)	2.15100	2.39000	2.62900	(10.0%)	1.000000	H12 || H17A
NOE[07] =	2.636882	-	(-)	2.34000	2.60000	2.86000	(10.0%)	1.000000	H13B || H18
NOE[08] =	3.053332	-	(-)	2.50200	2.78000	3.05800	(10.0%)	1.000000	H17A || H18
NOE[09] =	3.332277	0.021277	(10.7%)	2.70900	3.01000	3.31100	(10.0%)	1.000000	H17A || H20

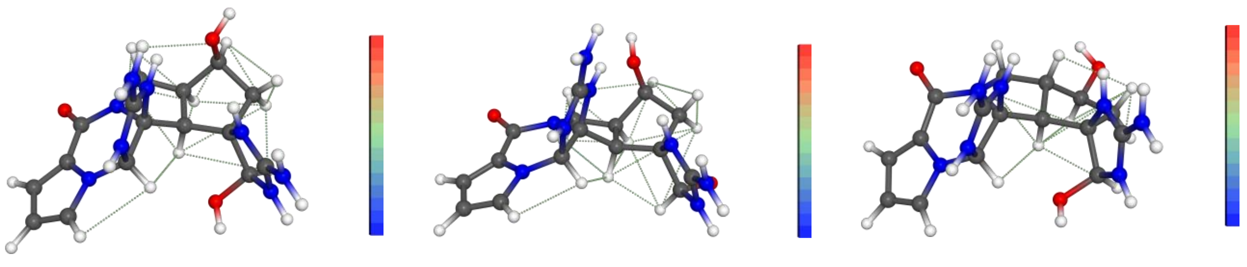

1a **1b** **1c**

Figure S1. Plot of the best-fit (minimum pseudo energy) DG structures of the palau'amine derivatives **1a**-**c** with color-coded representation of all NOE contacts used in the configurational and conformational analysis. The color scale was adapted from calculated final NOE violations, ranging from -0.40 Å (blue) to +0.40 Å (red).

NOE and Structure Data for Plakilactone H (2)

The following Table S2 and Figure S2 show the experimental and back-calculated NOE data used for the configurational analysis of the plakilactone (**2**). The Table was generated directly from the output files for the *ConArch*+*/DG* best-fit (minimum pseudo energy) structure obtained from configurational and conformational analysis.

Table S2. NOE data used for **2**. The experimental data is listed as d_{mean}, and the allowed lower and upper bonds – here $d_{mean} \pm 10\%$ – are labeled as $d_{lower} \cdots d_{upper}$; the averaged distances back-calculated from *ConArch*+*/DG* best-fit (minimum pseudo energy) structure data are labelled $d_{averaged}$, and the corresponding residuals are listed only if this value falls out of range, i.e. $d_{averaged} < d_{lower}$ or $d_{averaged} > d_{upper}$; all distance are given in [Å].

```
            D(averaged)     Deviation    (Percent) D(lower)    D(mean)  D(upper) (+/-Range)       Weights     NOE Contact
NOE[01] =      2.644129             -    (     - )  2.47500    2.75000   3.02500  (  10.0%)      1.000000     H16A|H16B|H16C || H15B
NOE[02] =      2.913799     -0.074201    ( -12.2%)  2.98800    3.32000   3.65200  (  10.0%)      1.000000     H16A|H16B|H16C || H8
NOE[03] =      3.465938      0.011938    (  10.4%)  2.82600    3.14000   3.45400  (  10.0%)      1.000000     H3 || H11A|H11B
NOE[04] =      2.588254     -0.021746    ( -10.7%)  2.61000    2.90000   3.19000  (  10.0%)      1.000000     H3 || H5B
NOE[05] =      3.209843      0.074843    (  12.6%)  2.56500    2.85000   3.13500  (  10.0%)      1.000000     H3 || H13A
NOE[06] =      3.529531      0.009531    (  10.3%)  2.88000    3.20000   3.52000  (  10.0%)      1.000000     H3 || H12A|H12B|H12C
NOE[07] =      2.647202     -0.151798    ( -14.9%)  2.79900    3.11000   3.42100  (  10.0%)      1.000000     H3 || H14A|H14B|H14C
NOE[08] =      3.565704      0.023704    (  10.7%)  2.89800    3.22000   3.54200  (  10.0%)      1.000000     H3 || H6
NOE[09] =      2.845071             -    (     - )  2.41200    2.68000   2.94800  (  10.0%)      1.000000     H8 || H9A|H9B
NOE[10] =      2.955072             -    (     - )  2.88900    3.21000   3.53100  (  10.0%)      1.000000     H8 || H10A|H10B|H10C
NOE[11] =      2.470103             -    (     - )  2.08800    2.32000   2.55200  (  10.0%)      1.000000     H8 || H6
NOE[12] =      2.481681             -    (     - )  2.37600    2.64000   2.90400  (  10.0%)      1.000000     H7 || H5B
NOE[13] =      2.649766             -    (     - )  2.38500    2.65000   2.91500  (  10.0%)      1.000000     H7 || H9A|H9B
NOE[14] =      2.441980     -0.015020    ( -10.6%)  2.45700    2.73000   3.00300  (  10.0%)      1.000000     H7 || H15A
NOE[15] =      3.840827             -    (     - )  3.18600    3.54000   3.89400  (  10.0%)      1.000000     H7 || H10A|H10B|H10C
NOE[16] =      3.874147      0.090147    (  12.6%)  3.09600    3.44000   3.78400  (  10.0%)      1.000000     H7 || H16A|H16B|H16C
NOE[17] =      3.102404             -    (     - )  2.64600    2.94000   3.23400  (  10.0%)      1.000000     H7 || H6
NOE[18] =      2.352820     -0.050180    ( -11.9%)  2.40300    2.67000   2.93700  (  10.0%)      1.000000     H5B || H13A
NOE[19] =      2.903985     -0.003015    ( -10.1%)  2.90700    3.23000   3.55300  (  10.0%)      1.000000     H5B || H6
NOE[20] =      3.489705      0.145705    (  14.8%)  2.73600    3.04000   3.34400  (  10.0%)      1.000000     H5B || H14A|H14B|H14C
NOE[21] =      2.676060             -    (     - )  2.67300    2.97000   3.26700  (  10.0%)      1.000000     H15B || H6
NOE[22] =      3.807998      0.023998    (  10.7%)  3.09600    3.44000   3.78400  (  10.0%)      1.000000     H15A || H8
NOE[23] =      2.632971             -    (     - )  2.46600    2.74000   3.01400  (  10.0%)      1.000000     H9A|H9B || H10A|H10B|H10C
NOE[24] =      2.621216     -0.096784    ( -13.2%)  2.71800    3.02000   3.32200  (  10.0%)      1.000000     H15A || H16A|H16B|H16C
NOE[25] =      2.616184             -    (     - )  2.48400    2.76000   3.03600  (  10.0%)      1.000000     H12A|H12B|H12C || H11A|H11B
```

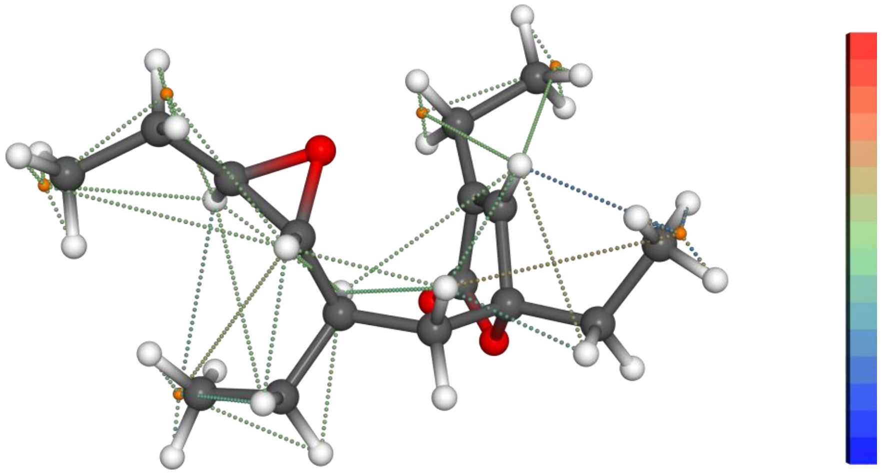

2

Figure S2. Plot of the best-fit (minimum pseudo energy) DG structures of plakilactone H (**2**) with color-coded representation of all NOE contacts used in the configurational and conformational analysis. The color scale was adapted from calculated final NOE violations, ranging from -0.40 Å (blue) to +0.40 Å (red).

NOE and Structure Data for Manzamine A (3)

The following Table S3-4 and Figure S3 show the experimental and back-calculated NOE and RDC data used for the configurational analysis of the manazamine A (**3**). The Table was generated directly from the output files for the *ConArch*+*/DG* best-fit (minimum pseudo energy) structure obtained from configurational and conformational analysis.

Table S3. NOE data used for **3**. The experimental data is listed as d_{mean}, and the allowed lower and upper bonds – here $d_{mean} \pm 10\%$ – are labeled as $d_{lower} \cdots d_{upper}$; the averaged distances back-calculated from *ConArch*+*/DG* best-fit (minimum pseudo energy) structure data are labelled $d_{averaged}$, and the corresponding residuals are listed only if this value falls out of range, i.e. $d_{averaged} < d_{lower}$ or $d_{averaged} > d_{upper}$; all distance are given in [Å].

	D(averaged)	Deviation	(Percent)	D(lower)	D(mean)	D(upper)	(+/-Range)	Weights	NOE Contact
NOE[01] =	2.476617	-	(-)	2.02700	2.25200	2.47700	(10.0%)	1.000000	H14A|H14B || H26
NOE[02] =	2.797847	-	(-)	2.61400	2.90400	3.19400	(10.0%)	1.000000	H17A|H17B || H26
NOE[03] =	2.497658	-	(-)	2.29800	2.55300	2.80800	(10.0%)	1.000000	H26 || H28A|H28B
NOE[04] =	2.570980	-	(-)	2.30500	2.56100	2.81700	(10.0%)	1.000000	H26 || H36A|H36B
NOE[05] =	2.159208	-	(-)	1.93200	2.14700	2.36200	(10.0%)	1.000000	H30A|H30B || H34
NOE[06] =	2.226290	-	(-)	1.99800	2.22000	2.44200	(10.0%)	1.000000	H32 || H33
NOE[07] =	2.742605	-	(-)	2.40400	2.67100	2.93800	(10.0%)	1.000000	H33 || H35A|H35B
NOE[08] =	2.449948	-	(-)	2.02600	2.25100	2.47600	(10.0%)	1.000000	H33 || H36A|H36B
NOE[09] =	2.558147	-	(-)	2.28000	2.53300	2.78600	(10.0%)	1.000000	H34 || H35A|H35B

Table S4. RDC data used for **3**. The experimental data is listed as D_{exp}, and the RDCs back-calculated from the structure model are labeled D_{calc}. All values including the Monte-Carlo derived error esitiomates are given in [Hz].

Results for Multi-Parameter SVD Fit of Calculated and Experimental Data:

	D(calc) [HZ]	+/- Error	D(exp) [Hz]	+/- Error	Rel. Weights		D(exp)-D(calc)		Normalized Weights	Atom Labels
D[01] =	21.704870	1.245029	21.823309	0.500000	1.000000	r[01] =	0.118439	w[01] =	0.035714	C3-H3
D[02] =	0.137062	1.671443	0.091663	0.500000	1.000000	r[02] =	-0.045399	w[02] =	0.035714	C4-H4
D[03] =	21.655839	1.096703	21.786998	0.500000	1.000000	r[03] =	0.131160	w[03] =	0.035714	C5-H5
D[04] =	-26.334976	1.461292	-26.566153	0.500000	1.000000	r[04] =	-0.231177	w[04] =	0.035714	C6-H6
D[05] =	-6.583622	1.763345	-6.649118	0.500000	1.000000	r[05] =	-0.065496	w[05] =	0.035714	C7-H7
D[06] =	22.529338	1.052731	22.695334	0.500000	1.000000	r[06] =	0.165996	w[06] =	0.035714	C8-H8
D[07] =	1.945254	1.370447	1.886530	0.500000	1.000000	r[07] =	-0.058724	w[07] =	0.035714	C11-H11
D[08] =	22.670474	1.481094	22.847812	0.500000	1.000000	r[08] =	0.177337	w[08] =	0.035714	C13-H13A+C13-H13B
D[09] =	23.926041	1.398360	24.107193	0.500000	1.000000	r[09] =	0.181152	w[09] =	0.035714	C14-H14A+C14-H14B
D[10] =	9.241049	1.107958	9.331405	0.500000	1.000000	r[10] =	0.090356	w[10] =	0.035714	C15-H15
D[11] =	-27.621917	1.440511	-27.669637	0.500000	1.000000	r[11] =	-0.047719	w[11] =	0.035714	C16-H16
D[12] =	-39.585065	1.967031	-39.747489	0.500000	1.000000	r[12] =	-0.162423	w[12] =	0.035714	C17-H17A+C17-H17B
D[13] =	-40.930775	1.964931	-41.123129	0.500000	1.000000	r[13] =	-0.192354	w[13] =	0.035714	C18-H18A+C18-H18B
D[14] =	12.472912	1.653159	12.535599	0.500000	1.000000	r[14] =	0.062687	w[14] =	0.035714	C19-H19A+C19-H19B
D[15] =	40.418646	1.296505	40.598288	0.500000	1.000000	r[15] =	0.179643	w[15] =	0.035714	C20-H20A+C20-H20B
D[16] =	-9.743054	1.726061	-9.846516	0.500000	1.000000	r[16] =	-0.103462	w[16] =	0.035714	C22-H22A+C22-H22B
D[17] =	27.387241	1.667193	27.491775	0.500000	1.000000	r[17] =	0.104534	w[17] =	0.035714	C23-H23A+C23-H23B
D[18] =	8.335456	1.500358	8.231811	0.500000	1.000000	r[18] =	-0.103645	w[18] =	0.035714	C24-H24
D[19] =	-30.251677	1.408419	-30.525030	0.500000	1.000000	r[19] =	-0.273353	w[19] =	0.035714	C26-H26
D[20] =	-6.911463	1.824540	-6.919452	0.500000	1.000000	r[20] =	-0.007989	w[20] =	0.035714	C28-H28A+C28-H28B
D[21] =	5.251709	1.918694	5.197804	0.500000	1.000000	r[21] =	-0.053905	w[21] =	0.035714	C29-H29A+C29-H29B
D[22] =	9.627178	2.190704	9.674817	0.500000	1.000000	r[22] =	0.047639	w[22] =	0.035714	C30-H30A+C30-H30B
D[23] =	-22.660937	2.127970	-22.765273	0.500000	1.000000	r[23] =	-0.104337	w[23] =	0.035714	C31-H31A+C31-H31B
D[24] =	2.729423	1.233013	2.762687	0.500000	1.000000	r[24] =	0.033264	w[24] =	0.035714	C32-H32
D[25] =	8.710639	1.522551	8.639642	0.500000	1.000000	r[25] =	-0.070997	w[25] =	0.035714	C33-H33
D[26] =	-28.729280	1.548104	-28.870231	0.500000	1.000000	r[26] =	-0.140951	w[26] =	0.035714	C34-H34
D[27] =	4.847359	1.544533	4.742710	0.500000	1.000000	r[27] =	-0.104650	w[27] =	0.035714	C35-H35A+C35-H35B
D[28] =	33.787954	1.525093	33.909669	0.500000	1.000000	r[28] =	0.121715	w[28] =	0.035714	C36-H36A+C36-H36B

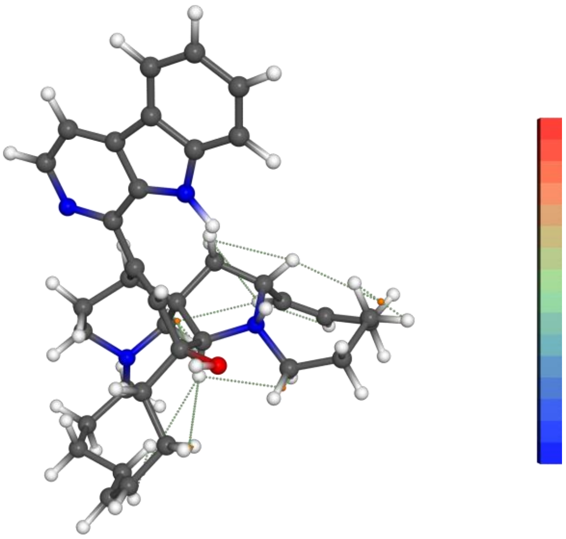

3 (NOEs)

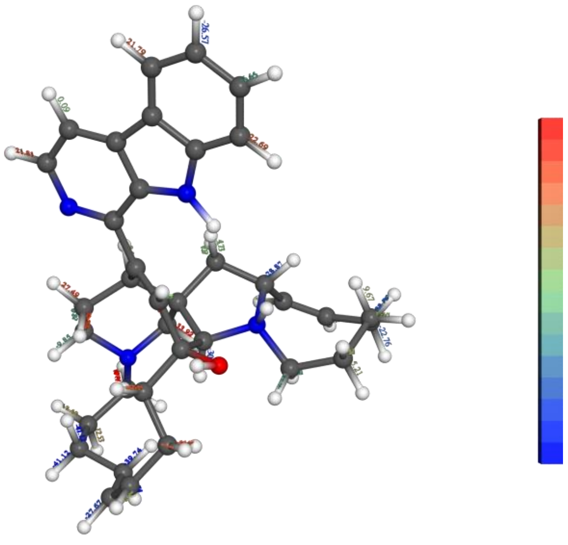

3 (RDCs)

Figure S3. Plot of the best-fit (minimum pseudo energy) DG structures of manzamine A (**3**) with color-coded representation of all NOE contacts (left model) and all RDCs (right model) used in the configurational and conformational analysis. The color scale was adapted from calculated final NOE violations, ranging from -0.40 Å (blue) to +0.40 Å (red), or to the range of RDCs (blue: -40 Hz, red: +40 Hz) back-calculated for **3**.

Atomic Coordinates for Compounds 1a-c, 2 and 3

The following tables provide the atomic coordinates for the final best-fit (minimum pseudo energy) DG structures of **1a-c**, **2**, and **3**, copy-and-paste for further usage:

Table S5. rDG best-fit structures (atomic coordinates) for compounds **1a-c**, **2**, and **3**.

**Palau'amine Derivatives**			**Plakilactone H**	**Manzamine A**
**1a**	**1b**	**1c**	**2**	**3**
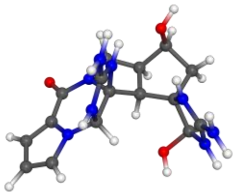	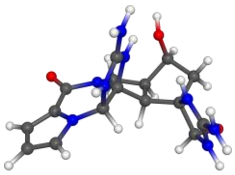	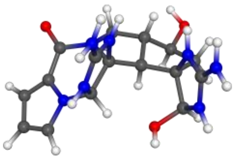	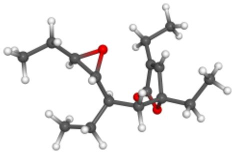	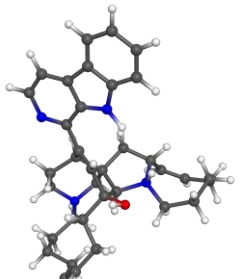

N1	-0.933681	2.737943	-0.294880
C2	-2.327504	2.652587	-0.295406
C3	-2.840589	3.937060	-0.124828
C4	-1.726753	4.828516	-0.017523
C5	-0.567493	4.077710	-0.123622
C6	-0.030870	1.651889	-0.442465
N7	0.789313	1.733518	-1.693290
C8	0.686092	0.616321	-2.439749
N8	1.321802	0.435268	-3.605882
N9	-0.133854	-0.278709	-1.857730
C10	-0.698665	0.231364	-0.543149
C11	-0.518545	-0.776397	0.624302
C12	-1.612086	-1.784176	0.465759
C13	-2.736412	-1.118413	-0.297383
N14	-2.164012	0.239200	-0.625665
C15	-3.011129	1.367291	-0.457015
O15	-4.226925	1.218094	-0.454890
C16	0.777670	-1.498804	1.095579
C17	0.308559	-3.048964	1.092420
C18	-0.892333	-3.107023	0.063254
O19	-0.330984	-3.095376	-1.235419
C20	1.270668	-1.097048	2.537806
O20	0.614343	0.165685	2.918561
N21	2.690393	-0.870742	2.350390
C22	3.068106	-1.004145	1.052250
N22	4.320063	-0.818670	0.617529
N23	2.014157	-1.342081	0.296894
H3	-3.901094	4.185663	-0.084505
H4	-1.771515	5.908476	0.123135
H5	0.480699	4.375113	-0.092890
H6	0.679798	1.700390	0.382579
H7	1.353203	2.536880	-1.936675
H9	-0.354584	-1.137093	-2.210907
H11	-0.770016	-0.171985	1.495824
H12	-2.003670	-2.049165	1.447870
H18	-1.533084	-3.968626	0.250814
H19X	-0.207042	-3.997763	-1.581631
H20	0.987717	-1.815280	3.307339
H20X	1.319692	0.768939	3.216970
H21	3.334601	-0.589670	3.077442
H23	2.071005	-1.521495	-0.684135
H8A	1.930731	1.154593	-3.994877
H8B	1.215913	-0.423623	-4.138617
H13A	-3.028787	-1.590808	-1.235178
H13B	-3.638027	-0.929405	0.285255
H17A	1.108561	-3.672912	0.693941
H17B	0.020103	-3.380568	2.089883
H22A	5.070192	-0.561122	1.258067
H22B	4.565686	-0.927383	-0.364095

N1	2.091626	-2.095249	-0.009804
C2	1.399632	-3.308700	0.053678
C3	2.278764	-4.328880	-0.292816
C4	3.544757	-3.722375	-0.575477
C5	3.411441	-2.355607	-0.397425
C6	1.557447	-0.811682	0.268337
N7	2.057408	-0.214513	1.538476
C8	1.056191	0.123198	2.377653
N8	1.245803	0.674015	3.584357
N9	-0.140149	-0.170085	1.832234
C10	-0.020141	-0.778115	0.457323
C11	-0.843589	-0.004942	-0.624244
C12	-2.236049	-0.560565	-0.505546
C13	-2.061338	-2.032744	-0.157276
N14	-0.669448	-2.070468	0.406129
C15	-0.015474	-3.350665	0.434225
O15	-0.639248	-4.336910	0.753742
C16	-0.995040	1.551685	-0.821584
C17	-2.476491	1.808959	-0.275905
C18	-2.972564	0.485762	0.396232
O19	-2.549095	0.488727	1.744935
C20	-0.868580	2.025538	-2.316638
O20	-2.112162	2.650406	-2.743613
N21	0.201078	3.010494	-2.262097
C22	0.656261	3.205568	-0.999688
N22	1.624155	4.074233	-0.680134
N23	0.001144	2.411396	-0.142307
H3	2.025595	-5.388257	-0.334347
H4	4.456224	-4.237225	-0.879216
H5	4.133252	-1.546879	-0.511653
H6	1.830139	-0.166320	-0.566676
H7	3.040856	-0.085725	1.748604
H9	-0.986914	-0.006841	2.257314
H11	-0.382005	-0.334824	-1.554953
H12	-2.772041	-0.481047	-1.451320
H18	-4.051002	0.359608	0.300540
H19X	-3.273740	0.253002	2.351779
H20	-0.692370	1.202390	-3.009081
H20X	-2.712566	1.943920	-3.049317
H21	0.549019	3.527944	-3.057124
H23	0.192817	2.376127	0.837675
H8A	2.182720	0.870187	3.936546
H8B	0.462819	0.913316	4.185017
H13A	-2.738404	-2.404187	0.611952
H13B	-2.132644	-2.708002	-1.009944
H17A	-2.501009	2.614405	0.458094
H17B	-3.127523	2.085798	-1.105132
H22A	2.077139	4.646041	-1.391451
H22B	1.938862	4.192162	0.280521

N1	-1.127977	-2.573854	0.934884
C2	-0.273816	-3.554316	0.406062
C3	-0.458757	-4.723708	1.121287
C4	-1.456440	-4.463778	2.119301
C5	-1.852509	-3.143847	1.988028
C6	-1.239844	-1.239054	0.480210
N7	-2.447648	-0.974252	-0.340560
C8	-2.147452	-0.457724	-1.553608
N8	-3.070133	-0.142752	-2.471943
N9	-0.824863	-0.311977	-1.696237
C10	-0.049560	-0.754856	-0.467513
C11	0.896581	0.325551	0.076153
C12	2.216542	0.119818	-0.664618
C13	2.320984	-1.403755	-0.817553
N14	0.879807	-1.811077	-0.837763
C15	0.611939	-3.228758	-0.717870
O15	1.101660	-3.997646	-1.497506
C16	0.789896	1.889136	0.269271
C17	2.354568	2.262499	0.422314
C18	3.191248	0.954414	0.256298
O19	4.410537	1.288201	-0.385896
C20	0.009362	2.324988	1.560320
O20	-0.657141	1.214069	2.165883
N21	-0.932422	3.316265	1.051944
C22	-0.828296	3.484086	-0.286492
N22	-1.578716	4.322661	-1.006640
N23	0.141695	2.680985	-0.778374
H3	0.064500	-5.663721	0.946140
H4	-1.836780	-5.174476	2.853024
H5	-2.581081	-2.556796	2.547182
H6	-1.254990	-0.604647	1.366437
H7	-3.392020	-1.170422	-0.033366
H9	-0.383710	0.043431	-2.472547
H11	1.079151	0.023957	1.107564
H12	2.245751	0.629052	-1.627908
H18	3.335734	0.425540	1.198379
H19X	4.662123	0.615504	-1.043950
H20	0.686957	2.736387	2.308461
H20X	-1.544957	1.485340	2.467483
H21	-1.606439	3.813200	1.622028
H23	0.382644	2.664742	-1.748565
H8A	-4.065035	-0.281281	-2.294501
H8B	-2.808183	0.243891	-3.374368
H13A	2.864273	-1.746958	-1.697978
H13B	2.755156	-1.881391	0.060774
H17A	2.623272	2.929792	-0.396599
H17B	2.541822	2.765464	1.371030
H22A	-2.296136	4.902023	-0.570071
H22B	-1.462742	4.413916	-2.014490

C1	1.424495	0.714735	2.153258
C2	2.078856	0.667628	0.775509
C3	1.378837	1.423829	-0.027627
C4	0.176689	2.065965	0.738694
C5	-1.147993	1.410698	0.208329
C6	-1.285218	-0.124005	0.388406
C7	-1.227633	-0.790652	-1.015873
C8	-0.656363	-2.191297	-1.167335
C9	-1.113856	-3.094760	-2.327058
C10	-2.081511	-4.201027	-1.831142
C11	3.327075	-0.162759	0.564266
C12	4.592323	0.715242	0.325659
C13	0.158303	3.575032	0.495002
C14	1.122855	4.169717	-0.601417
C15	-2.632986	-0.517826	1.081117
C16	-2.681570	-1.948428	1.641498
O1	0.443413	1.714190	2.076345
O2	1.642172	0.078907	3.125915
O3	0.093860	-1.012653	-1.544005
H3	1.411739	1.742716	-1.069418
H5A	-1.998891	1.946106	0.629529
H5B	-1.165239	1.620939	-0.861064
H6	-0.447534	-0.525177	0.958885
H7	-2.033227	-0.544407	-1.707596
H8	-0.399640	-2.688698	-0.232035
H9B	-0.228583	-3.539513	-2.781577
H9A	-1.606233	-2.463697	-3.066940
H10A	-2.752945	-3.815329	-1.063996
H10B	-2.687644	-4.576428	-2.655629
H10C	-1.532335	-5.040990	-1.405740
H11A	3.176275	-0.801371	-0.306098
H11B	3.479779	-0.806265	1.430684
H12A	4.349198	1.585637	-0.283769
H12B	5.002486	1.059731	1.274972
H12C	5.358387	0.136782	-0.190703
H13A	-0.818118	3.762910	0.048457
H13B	0.272368	4.130857	1.425672
H14A	2.203332	4.235340	-0.473495
H14B	0.657511	3.943617	-1.560809
H14C	0.770014	5.201028	-0.601228
H15B	-2.857561	0.214199	1.856878
H15A	-3.397820	-0.405063	0.312734
H16A	-3.504392	-2.083625	2.343485
H16B	-2.832144	-2.687012	0.854147
H16C	-1.782441	-2.197742	2.204977

C1	-2.335033	2.062666	-1.148102
N2	-3.505054	1.693042	-1.749376
C3	-4.269538	2.635835	-2.403261
C4	-3.904867	3.968770	-2.486401
C4A	-2.721391	4.379548	-1.882836
C4B	-2.023889	5.653419	-1.776599
C5	-2.300546	6.945055	-2.250084
C6	-1.379288	7.964439	-1.969870
C7	-0.199727	7.703196	-1.235139
C8	0.090763	6.414201	-0.757099
C8A	-0.833485	5.393043	-1.036008
C9A	-1.908677	3.415106	-1.198194
N9	-0.782275	4.037496	-0.692979
C10	-1.533443	1.001759	-0.466170
C11	-0.962786	1.214901	0.727282
C12	-0.137747	0.181219	1.490806
O12	0.703996	0.876225	2.428788
C13	-1.021769	-0.763674	2.348864
C14	-0.244057	-1.398398	3.571198
C15	-1.094043	-2.419699	4.305800
C16	-1.368048	-3.675052	3.915640
C17	-0.863070	-4.328092	2.626184
C18	-2.057271	-4.667602	1.721332
C19	-1.664145	-5.361467	0.391145
C20	-0.640583	-4.599258	-0.463872
N21	-0.914430	-3.138805	-0.505470
C22	-2.254331	-2.783586	-1.018615
C23	-2.531413	-1.298050	-0.708421
C24	-1.386895	-0.347379	-1.171461
C25	0.067955	-0.903044	-0.953107
C26	0.704343	-0.685285	0.477796
N27	2.079413	-0.021302	0.228216
C28	3.115774	-0.561438	1.191375
C29	4.370215	0.335426	1.343501
C30	5.199954	0.577838	0.027625
C31	5.607876	-0.590403	-0.770412
C32	4.583083	-1.452247	-1.455369
C33	3.276212	-1.277027	-1.698027
C34	2.405076	-0.091476	-1.283770
C35	1.015762	-0.094742	-1.895267
C36	0.100254	-2.414372	-1.284036
H3	-5.189529	2.305546	-2.885574
H4	-4.561417	4.645963	-3.032709
H5	-3.199049	7.161556	-2.827942
H6	-1.578409	8.968584	-2.344228
H7	0.489707	8.528372	-1.056628
H8	1.011548	6.232973	-0.202652
H9	0.034589	3.547823	-0.108228
H11	-1.094913	2.184836	1.206727
H12	-0.034294	1.384420	2.893263
H13A	-1.384556	-1.582479	1.727540
H13B	-1.864285	-0.205091	2.756605
H14A	0.646918	-1.888447	3.178621
H14B	0.028597	-0.577670	4.234647
H15	-1.537874	-2.101541	5.249140
H16	-2.010108	-4.232782	4.597399
H17A	-0.188250	-3.665756	2.083941
H17B	-0.344933	-5.257373	2.862978
H18A	-2.715092	-5.369475	2.233919
H18B	-2.574341	-3.746202	1.453449
H19A	-1.219963	-6.335742	0.595082
H19B	-2.548318	-5.478295	-0.235504
H20A	0.368423	-4.744591	-0.078016
H20B	-0.685024	-4.926001	-1.502796
H22A	-3.006769	-3.398808	-0.525220
H22B	-2.324785	-2.980004	-2.088454
H23A	-2.674753	-1.207815	0.368339
H23B	-3.448720	-1.012174	-1.223128
H24	-1.565399	-0.254921	-2.242763
H26	0.813714	-1.652587	0.968168
H27	2.072956	1.058895	0.482505
H28A	2.652552	-0.692505	2.169305
H28B	3.442090	-1.518989	0.785526
H29A	4.056084	1.311923	1.712111
H29B	5.010167	-0.174308	2.063732
H30A	4.617540	1.258988	-0.592800
H30B	6.109852	1.077135	0.360624
H31A	6.162899	-1.253077	-0.106400
H31B	6.274387	-0.243471	-1.560033
H32	4.978006	-2.382524	-1.863683
H33	2.801564	-2.097816	-2.235719
H34	3.046039	0.736949	-1.585397
H35A	0.640548	0.926076	-1.967680
H35B	1.012910	-0.517675	-2.899867
H36A	1.064391	-2.840967	-1.007347
H36B	-0.076012	-2.575719	-2.347520
